# 2400 How have characteristics of end-of-life family caregiving changed from 1999 to 2015? Preliminary results from two waves of nationally representative data

**DOI:** 10.1017/cts.2018.286

**Published:** 2018-11-21

**Authors:** Judith Vick, Jennifer Wolff

## Abstract

OBJECTIVES/SPECIFIC AIMS: Family members are often critical in the delivery of hands-on care and decisions about care for persons approaching end-of-life (EOL). Prompted by concerns about the poor quality and high costs of care at the EOL, recent delivery reform efforts—such as the growth of hospice and palliative care—have been directed at improving EOL care for both patients and family. Trends of the characteristics of EOL family caregivers and care recipients over time have not been well described. The goal of this study is to evaluate changes in EOL family caregiving from 1999 to 2015. METHODS/STUDY POPULATION: This study uses reconciled data from two nationally representative surveys and their linked caregiver surveys: the 1999 wave of the National Long-Term Care Survey (NLTCS) and the Informal Care Survey (ICS), and the 2015 wave of the National Health and Aging Trends Study (NHATS) and the National Survey of Caregiving (NSOC). RESULTS/ANTICIPATED RESULTS: Crude analysis shows that older adults living in the community and receiving help from family caregivers in the last year of life were significantly better educated (72% with greater than 12 years of education vs. 46%), and more diverse (78% White vs. 89%) in 2015 compared with 1999. Family caregivers in the last year of life were less likely to be female in 2015 compared with 1999 (74% vs. 68%, NS) and significantly less likely to be spouses (45% vs. 38%) in 2015. In 2015, a significantly greater proportion of older adults received help with five or more activities of daily living (47% vs. 34%), but family caregivers reported significantly lower levels of caregiving-associated distress: financial strain (80% reporting none in 2015 vs. 53%), emotional (51% vs. 39%), and physical strain (70% vs. 45%). In addition, a significantly greater proportion of EOL family caregivers used respite care in 2015 compared to 1999 (15% vs. 4%). DISCUSSION/SIGNIFICANCE OF IMPACT: Changes in the experience of EOL family caregiving may be impossible to capture in studies of single interventions, but tracking nationally representative trends can be used as an indicator of broader changes that take place cumulatively over time. Although studies of this nature cannot identify causal mechanisms of change, they are important to monitor long-term impact of program implementation and to guide future research, policy, and resource allocation.

